# A vicious cycle of fear of falling avoidance behavior in Parkinson’s disease: A path analysis

**DOI:** 10.1016/j.prdoa.2021.100089

**Published:** 2021-02-04

**Authors:** Merrill R. Landers, Kameron M. Jacobson, Nicole E. Matsunami, Hannah E. McCarl, Michelle T. Regis, Jason K. Longhurst

**Affiliations:** a University of Nevada, Las Vegas, United States; b Cleveland Clinic Lou Ruvo Center for Brain Health

**Keywords:** Postural balance, Gait, Falls, Postural instability, Balance confidence, Fear of falling, Avoidance behavior

## Abstract

**Background:**

Postural instability (PI) in Parkinson’s disease (PD) is associated with several negative downstream consequences.

**Objective:**

The purpose was to explore the validity of a theoretical model of these downstream consequences arranged in a vicious cycle wherein PI leads to decreased balance confidence, which in turn leads to increased fear of falling (FOF) avoidance behavior, which in turn leads to decreased physical conditioning, which then feeds back and negatively affects PI.

**Methods:**

A path analysis of cross-sectional data from 55 participants with PD was conducted. The four constructs in the model connected in succession were: 1. PI (principal components analysis (PCA) composite of the Unified Parkinson’s Disease Rating Scale PI and Gait Difficulty score, Timed Up and Go test, and Berg Balance Scale); 2. balance confidence (Activities-Specific Balance Confidence Scale); 3. FOF avoidance behavior (PCA composite of the FOF Avoidance Behavior Questionnaire and average number of steps per day); and, 4. physical conditioning (2-Minute Step Test).

**Results:**

The path model was an excellent fit to the data, χ2 (7) = 7.910, p = .341, CFI = 0.985, TLI = 0.968, RMSEA = 0.049 (90% CI: 0.000 to 0.179). The moderate to strong and uniformly significant parameter estimates were −0.519, −0.651, −0.653, and −0.570, respectively (ps < 0.01).

**Conclusions:**

PI directly and inversely predicted balance confidence, which in turn directly and inversely predicted FOF avoidance behavior. Furthermore, FOF avoidance behavior directly and inversely predicted physical conditioning, which directly and inversely predicted PI, thereby closing the cycle. These findings highlight the downstream consequences of PI in PD and support the notion of a vicious cycle of FOF avoidance behavior.

## 1 Introduction

Of the four cardinal signs in Parkinson’s disease (PD), postural instability (PI) is especially problematic because it can lead to falls, which in turn can hasten disability and mortality. A systematic review reported that falls are common in PD, with prevalence rates ranging from 35% to 90% [1]. Importantly, PI is of particular concern because it is progressive and can either be non-responsive to or poorly affected by PD medications [2], [3], [4]. Subsequently, as the disease progresses so too does the risk for falling [5], [6] and increased disability, both of which are natural consequences of disease-related reductions in mobility and other postural stability mechanisms [7]. Logically, PI and its sequelae are also associated with decreased balance confidence and heightened fear of falling (FOF) [8].

Decreased balance confidence is common in PD and is a natural consequence of PI. It can result from FOF, impaired balance, or impaired functional mobility, with impaired balance being the largest contributor to PI [9]. The interrelated concepts of balance confidence and FOF, though theoretically different [10] are complicated by contributing and contextual factors (e.g., anxiety, catastrophization). Gait and postural impairments have been shown to be large contributors to FOF in PD [8], [11], [12], [13], [14], [15], [16]. Despite this, as many as 75% of those who reported FOF did not report a recent history of falls [17]; thus, the development of FOF is complex and likely multifactorial. Regardless of the causative factors, FOF is common in PD with prevalence rates reported to range from 37% to 59% [11], [18], [19], [20], [21], [22]. When FOF, along with other factors, reaches a critical threshold or severity, it can begin to exert changes in daily function in the form of activity avoidance behavior [23].

Activity avoidance due to FOF is exhibited by up to 70% of individuals with PD [24], [25]. Avoidance behavior can be protective in that individuals may avoid activities that put them at risk of falling, which may limit the occurrence of falls in the short term. However, excessive avoidance behavior can have long term consequences as it has been shown to be associated with greater balance impairment, decreased balance confidence, and greater fall catastrophization, regardless of PD severity [23]. Ultimately, the downstream effects of avoidance behavior may hasten weakness and decrease physical conditioning [8]. This decreased physical conditioning can worsen PI by weakening already impaired balance systems, thus creating a vicious cycle. That is, a vicious cycle is one in which a chain of negative events reinforces themselves. In this case, it is postulated that the chain of negative events starts with PI and the downstream consequences then cycle back and reinforce the PI.

While all of the aforementioned evidence supports the face validity of this vicious cycle, there is no evidence in the literature that ties all of the relationships together into a vicious cycle. Therefore, the aim of this study was to explore evidence for the construct validity of the following proposed steps of this vicious cycle of FOF avoidance behavior in people with PD: 1. PI and subsequent gait and balance impairment directly contributes to reduced balance confidence and increased FOF; 2. decreased balance confidence and increased FOF increases avoidance behaviors; 3. avoidance behaviors lead to reductions in physical conditioning; and, 4. decreased physical conditioning further weakens already impaired balance systems and further increases PI and balance impairment. While we have hypothesized that these variables are linked in the aforementioned manner, the evidence from the literature only supports portions of these relationships. This is the first study to provide evidence in support of the construct validity of the theory underlying this self-reinforcing, vicious cycle of FOF avoidance behavior. Providing evidence for the validity of this cycle could contribute to physical therapy practice by highlighting the complexity of downstream consequences of PI in PD and identifying specific deleterious contributors that are potentially mitigable.

## 2 Materials and methods

### 2.1 Study design

A secondary analysis of a previously published cross-sectional research study was conducted for this study [23]. The original data collection comprised of an initial visit to participants’ homes to conduct performance testing, provide self-report questionnaires, and to apply activity monitors. A follow up visit was conducted one week later to collect the activity monitors and questionnaires. All data were collected from May 2011 to May 2013. Using these cross-sectional data, the vicious cycle of FOF avoidance behavior model was analyzed using path analysis.

### 2.2 Participants

Participants were recruited as a sample of convenience from PD support groups in the greater Las Vegas, Nevada, USA, area. A total of 59 individuals with a diagnosis of idiopathic PD (mean age = 72.0 ± 9.4 years; males = 45, females = 14; Hoehn and Yahr Scale score median = 2.0, mode = 3.0) participated in the study. Descriptive statistics of participant characteristics and relevant variables can be found in Table 1. Four participants were excluded from the analyses due to having missing data points on variables within the specified model, resulting in a final sample size of 55. The exclusion criteria were as follows: inability to read or speak English, cognitive impairment (defined as a score of < 24 on the Mini-Mental State Exam [MMSE]) [26], [27], or any non-PD-related comorbidities that significantly impaired balance. Written consent was obtained from all participants prior to data collection under [University of Nevada, Las Vegas] Biomedical Institutional Review Board approval. The sample size was estimated using the aims of the previous study; however, path analysis heuristics suggest that 5 to 10 participants per variable included in the model have been shown to be sufficiently robust [28]. Therefore, with a sample size of 55, the inclusion of four observed variables and no latent variables in the model was well within path analysis heuristics.

**Table 1:** Means, medians, proportions, and standard deviations for participant characteristics and relevant variables.

		PD Participants (n = 55)
Participant Characteristics		
	Age	72.09 ± 9.49
	Sex	41 male, 14 female
	Years from diagnosis	6.94 ± 4.40
	Socioeconomic status (median)	$50–75,000/year
	Education (median)	College: 4 years +
	Deep brain stimulation	Yes = 9, No = 46
	Falls in the last month	2.65 ± 12.06
	Falls in the last year	13.00 ± 50.00
	Injurious Falls in the last year	0.58 ± 1.26
	Hoehn and Yahr	1.0 = 13 2.0 = 13 3.0 = 25 4.0 = 1 5.0 = 1
	MDS-UPDRS part I (Mentation, Behavior and Mood)	14.46 ± 7.05
	MDS-UPDRS part II (Activities of Daily Living)	18.31 ± 8.67
	MDS-UPDRS part III (Motor Examination)	31.57 ± 15.45

Variables in substantive analyses		
	MDS-UPDRS PIGD sub score	1.22 ± 0.79
	Berg Balance Scale	44.05 ± 10.56
	Timed Up and Go	16.22 ± 19.21
	Activities Specific Balance Confidence Scale	63.26 ± 22.90
	Fear of Falling Avoidance Behavior Questionnaire	19.85 ± 12.47
	Daily number of steps taken	4622.99 ± 3383.04
	Two Minute Step Test	50.24 ± 35.89

### 2.3 Vicious cycle of FOF avoidance behavior model

**Fig. 1:**
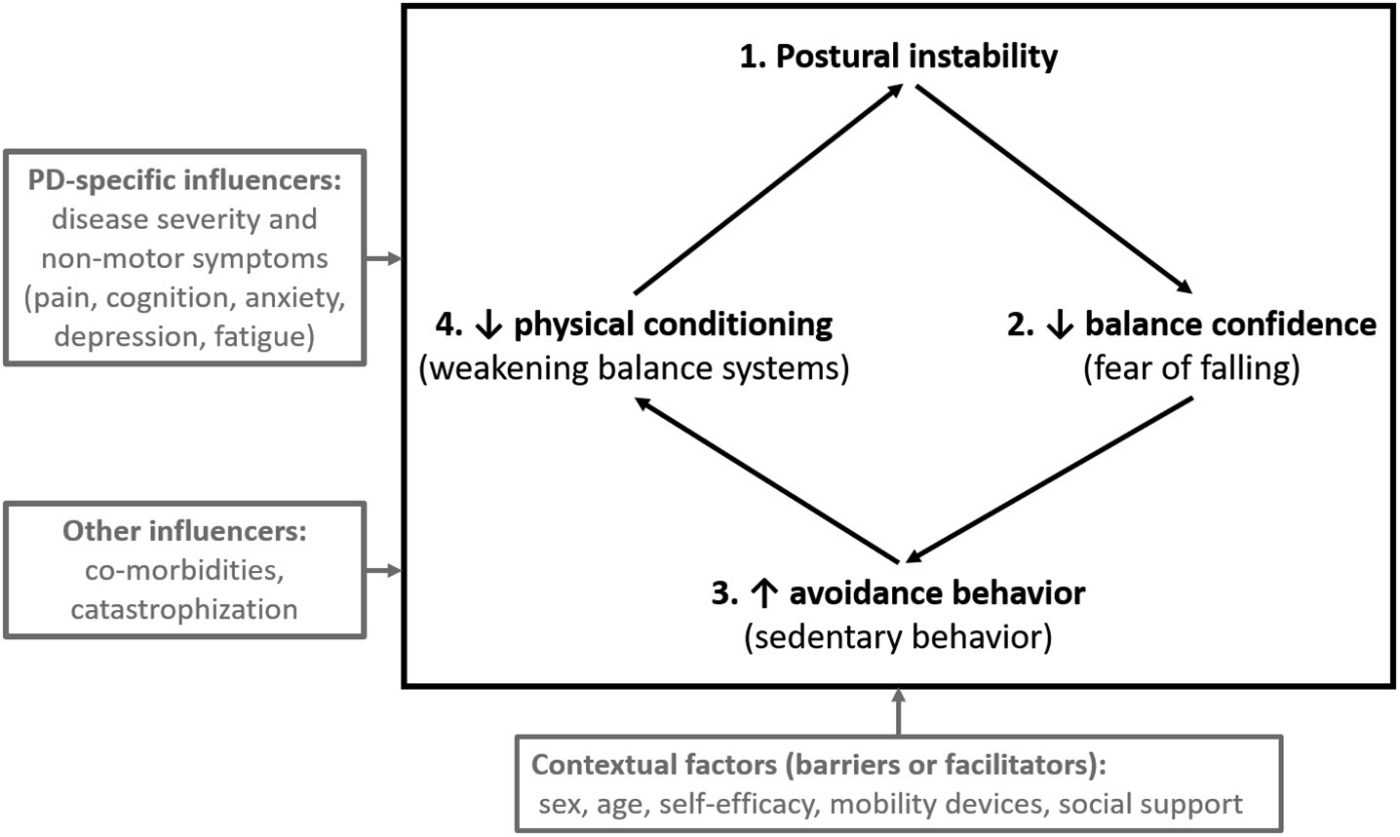
Theoretical model for the vicious cycle of fear of falling avoidance behavior in Parkinson’s disease.

The proposed model has four main constructs that are hypothesized in the following sequence: 1. PI; 2. balance confidence; 3. FOF avoidance behavior; and, 4. physical conditioning. The basic hypothesis proposes a causative relationship of the elements in that PI leads to a decrease in balance confidence, which in turn increases FOF avoidance behavior, which in turn decreases physical conditioning. The last element of the model is that decreased physical conditioning leads to more PI thereby closing the loop and starting a vicious cycle (Fig. 1). This model is intended to capture the main structural constructs of the model as a proof of concept. There are many other potentially contributing influencers and contextual factors (Fig. 1); however, they are not the main structural elements of the proposed vicious cycle. The variables used in each of the four main vicious cycle constructs are detailed below:

*Postural instability:* PI is a construct that essentially means a decrease in gait and balance function. To measure this construct, three different variables were combined into a composite measure (detailed in data analysis section): 1. a measure of disease specific PI (Movement Disorders Society Unified Parkinson’s Disease Rating Scale – PI and Gait Difficulty subscore (MDS-UPDRS PIGD subscore)) [29]; 2. a performance-based measure of gait (Timed Up and Go (TUG) test) [30]; and, 3. a performance-based measure of balance (Berg Balance Scale (BBS)) [31]. There is good evidence for the reliability and validity of the MDS-UPDRS and the PIGD subscore in PD [29], [32]. There is good evidence for the reliability of the TUG [33], [34] and its validity in PD [35]. There is also good evidence for the reliability of the BBS [33], [36] and its validity in PD [35], [37].
*Balance confidence:* The construct of balance confidence was measured using the Activities-Specific Balance Confidence (ABC) Scale [38]. There is good evidence for its reliability [33], [39] and validity [40] in PD populations.
*FOF avoidance behaviour:* To measure the construct of avoidance behavior, two different variables were combined into a composite score: 1. a self-report of FOF avoidance behavior (FOF Avoidance Behavior Questionnaire (FFABQ)) [41]; and, 2. an objective measure of physical activity levels (average number of steps per day) using activPAL monitors (PAL Technologies LTD, Glasgow, United Kingdom). Thus, the composite score of FOF avoidance behavior would include a self-report of avoidance behavior and also data from activity monitors which would act as a surrogate for sedentary behavior. There is good evidence for the reliability and validity of the FFABQ in neurologic populations, including PD [41] and also the activPAL in adult populations [42], [43]. Participants wore these activity monitors for at least 1 week with the first, last, and incomplete wear days excluded from the analysis. Data from all remaining usable days were averaged.
*Physical conditioning:* The construct of physical conditioning was measured using the 2-Minute Step Test (2MST) [44]. The 2MST has good reliability in PD populations [44] and has evidence for its validity as a measure of physical conditioning [45]. It is considered a suitable substitute for the 6MWT [46].

### 2.4 Procedural elements to control bias

Several precautions were taken to minimize risk of bias in the original study design. Testing was conducted in the homes of participants to provide a familiar environment and the selection of tests and measures was guided in part by the feasibility of conducting them in that setting. Additionally, testing took place in the “on” PD medication state (45 to 60 min after participants had taken their PD medication), and the duration of testing ranged from 45 to 75 min in length to ensure that participants remained in the “on” medication state throughout the session. At the end of the initial visit, questionnaires were reviewed item by item by the assessors to ensure responses were complete and appropriate. Additionally, participants were asked if they had questions about any of the items.

### 2.5 Data analysis

The data were analyzed to assess the quality of fit of the theoretical model described above. The analyses were completed using SPSS 24.0 (IBM SPSS Statistics for Windows, Armonk, New York, USA: IBM Corp) and Mplus 8.4 (Mplus for Macintosh, Los Angeles, California, USA: Muthén & Muthén) with α = 0.05. The analyses took place in two stages. First, principal components analyses (PCA) [47] were conducted in order to establish variable weights and create composite variables for PI and FOF avoidance behavior constructs. PCA was selected as the analysis method for creating composite variables as the variables included in each PCA were highly correlated [48]. No rotation was utilized during these analyses. As described above, the variables included in the PCA for PI were MDS-UPDRS PIGD sub-score, TUG, and BBS representing disease-related PI, gait-related PI, and balance-related PI, respectively. Variables included in the PCA for FOF avoidance behavior were the FFABQ and number of steps taken per day representing perceived avoidance behavior and level of activity, respectively. BBS and steps taken per day variables were inversely transformed. Steps per day was included as it was anticipated to be inversely related to the amount of avoidance behavior, as those who avoided functional activities were expected to take fewer steps per day. The first principle components were extracted for each construct (i.e., PI, FOF avoidance behavior) and used as composite variables in the subsequent analyses.

The second phase of analyses involved structural equation modeling with observed variables (i.e., path analysis) [49]. The target path model was specified to test the proposed model of the relationships among PI, balance confidence, FOF avoidance behavior, and physical conditioning. Direct paths from PI to balance confidence, from balance confidence to FOF avoidance behavior, from FOF avoidance behavior to physical conditioning, and from physical conditioning to PI were specified (Fig. 1). The ABC and 2MST variables were standardized prior to these analyses. The target model was estimated using MLR estimation routine in Mplus 8.4. For model fit evaluation, an inclusive approach was used involving a consideration of fit indices and the theoretical consistency and admissibility of parameter estimates. As the χ2 can be oversensitive to minor model misspecifications given even moderate-sized samples and contains a restrictive hypothesis test (i.e., exact fit), three approximate fit indices were considered: Root Mean Square Error of Approximation (RMSEA), ≤0.050 and 0.080 for close and reasonable fit [50], [51], respectively; Comparative Fit Index (CFI); and Tucker Lewis Index (TLI), ≥0.900 and 0.950 for acceptable and excellent fit [52], [53], respectively. However, RMSEA tends to reject models with small degrees of freedom, and its confidence interval is sensitive to degrees of freedom and sample size [54].

## 3 Results

**Fig. 2:**
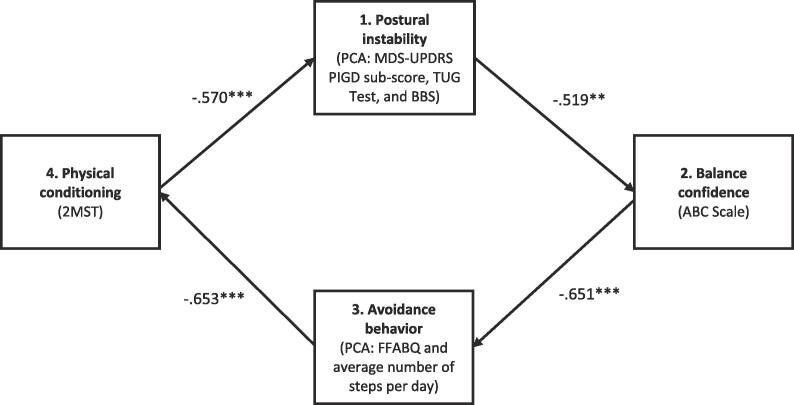
Retained path model with standardized estimates with main model constructs (outcome measures in parentheses). Abbreviations include the following: PCA (principal component analysis), MDS-UPDRS PIGD subscore (Movement Disorders Society Unified Parkinson’s Disease Rating Scale – Postural Instability and Gait Difficulty subscore), TUG (Timed Up and Go), BBS (Berg Balance Scale), ABC Scale (Activities-Specific Balance Confidence Scale), FFABQ (Fear of Falling Avoidance Behavior Questionnaire), and 2MST (2-Minute Step Test). *p < .05, **p < .01, ***p < .001.

The specified path model was an excellent fit to the data, χ2 (7) = 7.910, p = .341, CFI = 0.985, TLI, = .968, RMSEA = 0.049 (90% CI: 0.000 to 0.179). The final model with parameter estimates is shown in Fig. 2. In totality, the model explained 34.2% of the variance in balance confidence, 46.2% of the variance in FOF avoidance behavior, 48.4% of the variance in physical conditioning, and 37.0% of the variance in PI.

## 4 Discussion

Consistent with our original hypothesis, each of our four foundational constructs (PI, balance confidence, FOF avoidance behavior, and physical conditioning) were found to directly and inversely predict the next construct in a cyclical manner. Taken together, these statistical relationships highlight the potential downstream consequences of PI in PD and support the plausibility and validity of a vicious cycle of FOF avoidance behavior in PD as described in Fig. 1. Moreover, because of the strong associations and excellent fit found in the analyses, they support the relationship inferences of the model. To our knowledge, this is the first study to provide evidence for a balance-related, self-reinforcing, vicious cycle in older adults with PD as has been postulated by other researchers [8], [10], [23]. Moreover, these results are consistent with scientific data that supports some of the relationships among constructs of this theoretical model [16], [21], [24], [55], [56], [57]. However, we caution the interpretation and generalizability of the findings since this vicious cycle is likely more complex, involving other influencers and contextual factors. Further research to build out the model from these four foundational constructs is warranted.

Other influencers and contextual factors may also be involved either directly or indirectly as barriers or facilitators of the different components of the cycle. For instance, age [12], [58] gender [58] disease severity [24] and pain [58] have all been shown to be associated with different aspects of the cycle in people with PD. In addition, psychological comorbidities and PD sequelae have also been identified as contributors to FOF and FOF avoidance behaviors, including anxiety [23], [56], [59], [60], [61], catastrophization [23], [56], and depression [23], [62], [63]. Fatigue, a prominent non-motor symptom in PD, has also been shown to be associated with more sedentary behavior and poorer functional capacity [64] and may contribute to avoidance behavior as it has also been shown to be an explanatory factor for FOF [12]. Lastly, FOF in older adults has been associated with fewer social contacts and FOF avoidance behavior has been associated with a decrease in social support [60]. Social support has also been shown to be a predictor of physical activity in people with PD [58].

From a clinical perspective, if the main constructs in this framework are indeed ordered in the correct causative pathway, it presents an opportunity for clinicians to break up this vicious cycle by treating the mitigable factors. For instance, it has been shown that a demanding balance program not only improves balance, but also reduces FOF in people with PD [65], [66]. Furthermore, walking ability was found to be the largest contributor to FOF in patients with PD; therefore, improvements in this area may help to decrease FOF [15]. However, there is a lack of research regarding treatment for FOF avoidance behavior, though Nilsson et al suggested that interventions targeting FOF avoidance behavior should consider targeting FOF, pain, and walking difficulties [55]. Conradsson et al found that highly challenging balance training for people with PD compared to usual care was associated with improvements in gait and balance with promising trends in physical activity levels [67].

Limitations with the original study that carried over to the secondary analysis included a reduction in possible constructs secondary to material and space restrictions that were an artifact of testing at participants homes. Incongruities in steps per day data also existed due to duration of uninterrupted wear time, time of day, and part of the week from which usable data were extracted. Given the nature of the cross-sectional study design, data collection took place across one session, providing a snapshot that might not be a true reflection of the participants’ level of performance. Moreover, this design did not establish temporality for causal inference. However, establishing temporality of the factors would not have been feasible considering the overlap and time needed to develop the downstream effects. Due to sample size limitations, the decision was made to not include influencers and contextual factors outside of the main constructs of the proposed model. For the same reason, we did not investigate the role of falls in this study and it is a limitation of the proposed framework. The sample had a reasonable number of participants at low to moderate disease state (Hoehn and Yahr 1–3), but only two participants in advanced disease states (Hoehn and Yahr 4 and 5). This, coupled with the elimination of those with significant cognitive impairment, impacted the study’s generalizability to all with PD. While path analysis is useful in evaluating causal hypotheses and in estimating the significance of the causal connections between variables, it cannot prove causation and can only provide evidence to support causal inference. Using PCA adds additional limitations as it relies on variance for its principal components estimates and can bias the principal component toward the variable with highest variance. After applying a PCA, the original variables turn into principal components which are not as readable nor interpretable as the original variables [68].

## 5 Conclusion

PI directly and inversely predicted balance confidence, which in turn directly and inversely predicted FOF avoidance behavior. Furthermore, FOF avoidance behavior directly and inversely predicted physical conditioning, which directly and inversely predicted PI, thereby closing the cycle. These findings highlight the potential downstream consequences of PI in PD and support the notion of a vicious cycle of FOF avoidance behavior. While these data provide evidence for this self-reinforcing vicious cycle, the results of this study should be interpreted with some caution as the design and analysis are limited regarding causal inference. Further research is warranted to support the validity of this model, establish temporal relationships among the main constructs, and to explore other influencers and contextual factors that may contribute to the model.

## Funding sources and conflicts of interest

The research reported in this publication was supported by a University of Nevada, Las Vegas, Student Opportunity Research Award. All authors have contributed in a significant way as to merit authorship. None of the authors report any conflicts of interest and all have adhered to ethical standards stipulated in the Institutional Review Board approval which was received from the University of Nevada, Las Vegas Biomedical Institutional Review Board. All authors affirm the Journal’s Ethical Publication Guidelines. Other than the funding sources listed above, none of the authors have received financial or material support for this research and work. Dr. Landers is currently receiving financial support on projects unrelated to this line of research. Dr. Landers is a Co-Investigator on a project titled “Long-term transcranial direct current stimulation in Parkinson's disease,” which is funded by the 10.13039/100000065National Institute of Neurological Disorders and Stroke, NIH (R15NS098342).

## CRediT authorship contribution statement

**Merrill R. Landers:** Conceptualization, Methodology, Validation, Formal analysis, Investigation, Writing - original draft, Writing - review & editing, Project administration. **Kameron M. Jacobson:** Writing - original draft, Writing - review & editing. **Nicole E. Matsunami:** Writing - original draft, Writing - review & editing. **Hannah E. McCarl:** Writing - original draft, Writing - review & editing. **Michelle T. Regis:** Writing - original draft, Writing - review & editing. **Jason K. Longhurst:** Conceptualization, Methodology, Formal analysis, Writing - original draft, Writing - review & editing, Project administration.
